# Prevalence of musculoskeletal disorders among taxi drivers in Yaoundé, Cameroon: preventive effect of physical activity

**DOI:** 10.1186/s12891-022-05971-w

**Published:** 2022-11-26

**Authors:** Chrystelle Cassandre Ngatcha Tchounga, Marcel Azabji Kenfack, Wiliam Richard Guessogo, Jerson Mekoulou Ndongo, Elysée Claude Bika Léle, Clarisse Noël Ayina Ayina, Abdou Temfemo, Bienvenu Bongue, Samuel Honoré Mandengue, Laurent Serge Etoundi Ngoa, Peguy Brice Assomo Ndemba

**Affiliations:** 1 Department of Physiology, Faculty of Medicine and Biomedical Sciences, University of Yaoundé I, Yaoundé, Cameroon; 2 Department of Human and Social Sciences Applied to Physical Activities and Sports, National Institute of Youth and Sports, Yaoundé, Cameroon; 3 Physiology and Medicine of Physical Activities and Sports Unit, Faculty of Sciences, University of Douala, Douala, Cameroon; 4 Department of Biological Sciences, Faculty of Medicine and Pharmaceutical Sciences, University of Douala, Douala, Cameroon; 5 UMR 1059, INSERM, SAINBIOSE, Jean Monnet University, Saint Etienne, France

**Keywords:** Musculoskeletal disorders, Physical activity, Professional driver, Cameroon

## Abstract

**Background:**

Musculoskeletal Disorders (MSDs) are very common conditions in the workplace. Among professional drivers, there would be an increased risk of developing these disorders. Identifying the associated factors would allow us to better devise effective prevention strategies. Our objective was to determine the prevalence of MSDs among taxi drivers in the city of Yaoundé and to search for associated factors, mainly the level of physical activity.

**Methods:**

We conducted an analytical cross-sectional study of 151 adult male professional taxi drivers. We used a non-probabilistic consecutive and non-exhaustive sampling method. Sociodemographic, anthropometric and occupational data were collected. MSDs over the past 12 months were assessed using the Nordic Questionnaire and physical activity level was determined by the World Health Organization (WHO) Global Physical Activity Questionnaire (GPAQ). Univariate logistic regression models, followed by a multivariate logistic regression, were used to determine factors associated with the presence of MSDs.

**Results:**

The overall prevalence of MSDs was 86.8% (95% CI 80.8 – 91.4); the most affected areas were mainly the lower back (72.8%) the neck (42.4%), and the knees (29.1%). Job dissatisfaction was associated with MSDs (OR = 2.1 95%CI = 1.1–3.9). Most taxi drivers (62.9%) had a low physical activity level and no association was found between the physical activity level and MSDs.

**Conclusions:**

MSDs are common ailments among taxi drivers in Yaoundé (Cameroon). There is a need to think about how to address job dissatisfaction and better identify other associated factors in order to define good prevention strategies.

## Background

Musculoskeletal disorders (MSDs) are disorders of the musculoskeletal system resulting from a number of factors wherein the work environment and working contribute significantly in different measures to the cause of the disease [1]. They refer to a number of heterogeneous conditions presenting primarily with pain whose severity varies from mild periodic symptoms to severe chronic and debilitating conditions [2]. They are the most common pathologies encountered at the workplace. In Europe, out of every five workers, three complain of MSDs [3]. They cause an alteration in the quality of life, a drop in productivity at work, early withdrawal from the workplace, and represent a high cost for the health system. The United States estimates that approximately $45 billion is the annual expenditure related to this condition [4]. Moreover, they are ranked first among the conditions that lead to prolonged absenteeism from work [4]. In Africa, a prevalence range of 15–93% has been found depending on the field of work [5]. In Cameroon, in the health, railway, education, and handling sectors, a prevalence ranging from 74 to 100% [6–9] has been reported. In a study among nurses in the Fako division of the South West region of Cameroon, 50% of them stated that MSDs slowed down their activities at work, 37.5% declared that this led to a decrease in the efficiency of nursing care provided and 12.5% ​​had been absent from work because of the condition [10].

In developed countries, MSDs have been controlled thanks to a better assessment of the nature of work-related risk factors and of protective factors, which has led to good preventive measures [11]. In addition to the positive effects of physical activity on the primary and secondary prevention of many chronic and debilitating diseases such as diabetes, cardiovascular diseases, stroke, cancer, and depression [12, 13], some studies have shown the sure benefit of physical activity as a complementary modality for managing MSDs [14–16]. Other studies have suggested that regular physical activity is associated with a lower incidence of MSDs in workers [17, 18]. This would therefore imply that an optimal level of physical activity could be an effective primary and secondary prevention strategy.

In all known fields of work, professional driving has been described as being associated with an increased risk of MSDs compared to the general population [19]. Indeed, a prolonged sitting position, the car’s vibrations, a poor posture, the carrying of loads, and work-related stress have been identified as factors favoring the occurrence of these disorders [20]. Worldwide, a prevalence ranging from 51–93% has been reported among professional drivers [21]. Few studies have been carried out in sub-Saharan Africa to assess the prevalence of MSDs in this occupation, let alone the associated factors whose identification would help to devise effective prevention strategies based on empirical data. We therefore proposed to determine the prevalence of MSDs within a population of professional drivers, in this case, taxi drivers in the city of Yaoundé, as well as their physical activity level, and to look for an association between the two primarily, and secondarily look for other associated factors.

## Methods

### Participants

This cross-sectional study was conducted between February 2021 and June 2021 at various taxi drivers Union and taxi stands in Yaoundé, the capital city of Cameroon. All the participants recruited in the study were male drivers with at least one year experience of driving taxi, who had no history of traumatic road or work accidents and aged 21 years or more.

### Procedure

The participants in this study were contacted at the headquarters of the drivers’ Union while doing their meetings. All consented participants were evaluated using a five-part questionnaire that included the Nordic Musculoskeletal Questionnaire (NMQ). In all, a total of 151 had completed and evaluable questionnaire by a face-to-face interview.

The study participants were recruited through their respective unions. These unions organize regular meetings between their members and it was during these meetings that we collected the data. The data collection form included a questionnaire on socio-demographic characteristics, occupational characteristics of the taxi driver, MSD assessment, level of physical activity and anthropometric parameters. Socio-demographic and occupational characteristics were collected through questions. The assessment of MSDs was done with the Nordic questionnaire proposed in 1987 by Kuorinka, I. and subsequently adapted. It was designed to answer the question: "Do musculoskeletal disorders occur in a given population, and if so, in which parts of the body are they located? The questionnaire presents a figure of the human body with nine anatomical regions (three in the upper limbs, three in the lower limbs and three in the trunk) in order to help the subject being assessed to answer "yes" or "no" regarding the presence or absence of MSD symptoms for the different regions of the body over the last 12 months and then over the last 07 days, as well as the impact on work activity. It has been documented as having acceptable validity and reliability [22, 23]. It is widely used in occupational medicine to assess MSDs. The physical activity level of the taximen was assessed by the Global Physical Activity Questionnaire (GPAQ) developed by the WHO. It collects information on physical activity in the following three situations (or domains): activities at work, moving from one place to another, leisure activities and by means of algorithms to determine the physical activity level of an individual The questionnaire has been tested and found to be valid and reliable for epidemiological purposes in adults [24]. Weight and height were measured to assess weight status through the calculation of the body mass index.

### Statistical analysis

Data entry was done in Cs Pro 7.5 and analysis in SPSS (Statistical Package for the Social Sciences) 20.0. Prevalence’s were reported as percentages with 95% confidence intervals. To search for factors associated with MSDs, Univariate logistic regression models has been done primarily followed by a multivariate logistic regression with all the variable that had association with p value < 0.2, Chi-square test was used to check the significance. Fischer test was used when one or more of the numbers in the contingency table were less than five. Significant differences were considered at last for a *p*-value < 0.05.

### Ethical consideration

Following administrative procedures that resulted in the issuance of an ethical clearance No. 2073/CSRDHRC/2021 from the Regional Research Ethics Committee for Human Health of the Centre and in obtaining research authorizations from the presidents of the taxi drivers' unions, the research was conducted in accordance with the ethical principles of the European Union. The study was carried out in strict compliance with the fundamental principles of medical research according to Helsinki’s Declaration.

## Results

The average age of the taxi drivers who participated in our study was 39.3 ± 8.1 years with a minimum of 23 years and a maximum of 58 years. The most represented age group was that of [30–40[ years with a percentage of 41.7%. In our study population, nearly two-third of the participants had a high school education level. The majority of participants (45.7%) were overweight and 27.8% were obese. Among the taxi drivers participating in the study, 43% had been in this profession for more than 15 years. The working hours varied from 3 to 20 h, with an average of 9.2 ± 2.4 h. These taxi drivers cumulatively worked 21 to 140 h weekly, with an average of 56.9 ± 17.6 h. More than half of the taxi drivers had a low level of physical activity (62.9%). Almost a third had a moderate level of physical activity (30.5%) and only 6.6% had a vigorous level of physical activity (Table 1).

**Table 1:** Sociodemographic and work related information on the surveyed taxi drivers

Variables	Counts	Percentages
**Age (in years)**		
[20–40[	78	51.6
[40–60[	73	48.3
**Matrimonial Status**		
Married	124	82.1
Single	27	17.9
**Literacy level**		
Unschooled and Primary school	48	31.8
Secondary and High school	96	63.6
University	7	4.6
**Weight Status**		
Normal	40	26.5
Overweight	69	45.7
Obesity	42	27.8
**Job Tenure (in years)**		
< 10	55	36.4
[10–15[	31	20.5
> 15	65	43
**Daily Number of working hours**		
≤ 6	66	43.7
7	85	56.3
**Weekly Number of working hours**	15	9.9
< 60	98	64.9
≥ 60	53	35.1
**Physical Activity Level**		
Low	95	62.9
Moderate	46	30.5
High	10	6.6

The overall prevalence of musculoskeletal disorders in the last 12 months was 86.8% (95% CI 80.8–91.4). Among the participants, 57% complained of musculoskeletal pain in the past seven days. Nearly a third (32.1%) of the subjects having had a complaint of pain in the last 12 months stated that they had stopped work earlier on certain days due to the pain. Finally, 20.6% of taxi drivers affected by MSDs were absent from work because of pain (Table 2).

**Table 2:** Distribution of participants according to reported MSDs symptoms and its impact on work for the past 12 months

Variables	Counts	Percentages
**In the last 12 months**		
Yes	131	86.8
No	20	13.2
**In the last 7 days**		
Yes	86	57
No	65	43
**Reduced working hours (*n* = 131)**		
Yes	42	32.1
No	89	67.9
**Absenteeism (*n* = 131)**		
None	104	79.4
More than one day	27	20.6

Participants with a low physical activity level had a greater proportion of people affected by MSDs but this difference was not significant. For the lower back and neck regions, we did not find a significant association between the physical activity level and MSDs (Table 3).

**Table 3:** Association between physical activity level and MSDs

Physical Activity Level	Presence of MSDs	Absence of MSDs	OR (CI at 95%)	*p*-value
**Overall**				
Low	84 (88.4)	11 (11.6)	1	
Moderate	38 (82.6)	8 (17.4)	0.6 (0.2 – 1.6)	0.343
High	9 (90)	1 (10)	1.1(0.1 – 10.2)	1.000
**Lumbar region**				
Low	74 (77.9)	21 (22.1)	1	
Moderate	32 (69.6)	14 (30.4)	0.6 (0.3 – 1.4)	0.292
High	8 (80)	2 (20)	1.3 (0.3 – 5.6)	0.706
**Neck and Nape**				
Low	40 (42.1)	55 (57.9)	1	
Moderate	20 (43.5)	26 (56.5)	1.1 (0.5 – 2)	0.877
High	4 (40)	6 (60)	1.1 (0.2 – 3.3)	1.000

Age over 39 years and obesity increased the risk of having MSDs in our study population, but these associations were not significant (*p* > 0.05).

Having more than 12 years of job tenure and the common habit of taking naps in the taxi increased the risk of having MSDs in our study population, but these associations were not significant (*p* > 0.05) (Table 4).

**Table 4:** Personals and professional characteristics and MSDs

	**Presence of MSDs**	**Absence of MSDs**	**OR (CI at 95%)**	***p*-value**
**Age (in years)**				
> 39	66 (90.4)	7 (9.6)	2 (0.71—5)	0.200
≤ 39	65 (83.3)	13 (16.7)	1	
**Matrimonial status**				
Married	108 (87.1)	16 (12.9)	1.2 (0.4 – 3.8)	0.791
Single	23 (85.2)	4 (14.8)	1	
**Literacy level**				
Secondary School level or lower	126 (87.5)	18 (12.5)	2.8 (0.5 – 15.5)	0.233
University	5 (71.4)	2 (28.6)	1	
**BMI**				
≥ 30	39 (92.9)	3 (7.1)	2.4 (0.7 – 8.7)	0.170
< 30	92 (84.4)	17 (15.6)	1	
**Job tenure (in years)**				
˃ 12	69 (92)	6 (8.0)	2.5 (0.9–10)	0.059
≤ 12	62 (81.6)	14 (18.4)	1	
**Daily number of working hours**				
≥ 12	20 (83.3)	4 (16.7)	0.7 (0.2 – 2.4)	0.590
< 12	111 (87.4)	16 (12.6)	1	
**Number of working days per week**				
˃5	107 (86.3)	17 (13.7)	0.77 (0.2–3.3)	1.000
≤ 5	24 (88.9)	3 (11.1)	1	
**Weekly number of working hours**				
˃ 56	48 (90.6)	5 (9.4)	1.66 (0.59—5)	0.310
≤ 56	83 (84.7)	15 (15.3)		
**Carrying of Luggage**				
Many times per week	63 (85.1)	11 (14.9)	0.8 (0.3 – 1.9)	0.565
Rarely	68 (88.3)	9 (11.7)	1	
**Taking naps in the taxi**				
≥ once per week	25 (96.2)	1 (3.8)	4.5 (0.6 – 35.1)	0.201
Rarely	106 (84.8)	19 (15.2)	1	

Taxi drivers who were dissatisfied with their jobs were three times more likely to develop musculoskeletal disorders and this association was close to significance in bivariate analysis (OR = 3.3, 95% IC = 0.9–12.5) (Table 5).

**Table 5:** Workplace comfort, psycho-social factors and MSDs (self-reported)

	**Presence of MSDs**	**Absence of MSDs**	**OR (CI at 95%)**	***p*-value**
**Seat Comfort**				
Uncomfortable	15 (93.8)	1 (6.3)	2.5 (0.3–20)	0.697
Comfortable	116 (85.9)	19 (14.1)	1	
**Steering wheel comfort**				
Uncomfortable	7 (77.8)	2 (22.2)	0.5 (0.1–2.5)	0.340
Comfortable	124 (87.3)	18 (12.7)	1	
**Working State**				
Employee	77 (87.5)	11 (12.5)	1.1 (0.45–3.3)	0.750
Owner	54 (85.7)	9 (14.3)	1	
**Perceived Stress**				
Stressful	94 (86.2)	15 (13.8)	0.8 (0.3 – 2.5)	0.763
Not stressful	37 (88.1)	5 (11.9)	1	
**Job Satisfaction**				
Unsatisfied	49 (84.2)	3 (5.8)	3.3 (0.9–12.5)	0.050
Satisfied	82 (82.8)	17 (17.2)	1	

On multivariate analysis, job dissatisfaction clearly emerged as the sole factor significantly associated with MSDs (Table 6).

**Table 6:** Logistic regression analysis of sociodemographic and professional factors associated to MSDs

	**OR**	**95% CI**	***P* value**
**Job tenure (in years)**			
˃ 12	2.5	0.9–7.3	0.091
≤ 12		1	
**Job Satisfaction**			
Unsatisfied	**2.1**	**1.1–3.9**	**0.031**
Satisfied		1	
**BMI**			
≥ 30	0.4	0.1–1.7	0.232
< 30		1	

## Discussion

The objective of this study was to determine the prevalence of musculoskeletal disorders (MSDs) among taxi drivers in Yaoundé (Cameroon) and the factors associated to them. We carried out an analytical cross-sectional study and the prevalence found was 86.8%. The most affected area of ​​the body was the lower back. Job dissatisfaction was associated with a higher frequency of MSDs. Most taxi drivers (62.9%) had a low physical activity level and we did not find a significant association between the level of physical activity and MSDs.

The overall prevalence of MSDs among taxi drivers was 86.8%. This prevalence is found in the prevalence range of MSDs among professional drivers reported in the literature. Leonard et al. and Zulkarnain et al. in their respective reviews on MSDs in professional drivers found prevalences ranging between 43.1% and 93% [21, 25]. This large interval can be explained by the data collection method chosen. In some studies, participants having reported of pain or any other related complaint (limiting mobility or discomfort) frequently during the course of the year or who reported that the complaint lasted at least 24 consecutive hours were counted as having MSDs [26, 27]. In the above-mentioned studies, the overall prevalence was lower than in those which, like ours, took into consideration any occasional musculoskeletal symptom reported by the professional driver. Knowing that before becoming chronic, the clinical expression of MSDs vary greatly with time and the prognostic factors are poorly known, it seemed important to us to report all the possible cases [28]. Abledu et al. [29] using an approach identical to ours found a prevalence of 70.5% among taxi drivers in Ghana. Similarly, Akinpelu et al. [30] found a prevalence of 89.3% among professional drivers (bus, taxi) in Nigeria. These two results are similar to ours. The high prevalence found in our study as everywhere else confirms the hypothesis that professional drivers are at high risk of developing MSDs.

The back region was the most affected region as described in the literature [21]. In professional drivers, the back region would be the major site because they have many risk factors for spinal pain compared to the general population, mainly due to vehicle vibrations and also a sedentary lifestyle [31]. Furthermore, in our study, we found a prevalence of 72.8%, which is higher than the 56% estimated in a meta-analysis carried out by Leonard et al. in a systematic review on the prevalence of MSDs among professional drivers in 04 continents [21]. Our result was different from those found in Ghana and Nigeria where their prevalence were 34.3% and 30% respectively [29, 30]. This difference can be explained by the fact that there were differences in the job tenures of the professional drivers in the different study populations. In the study carried out in Ghana, less than 2% of taxi drivers had worked for more than 12 years; in Nigeria, only 25% of the professional drivers had a job tenure of more than 15 years. Conversely, in our study, almost half of the taxi drivers (43%) had worked for more than 15 years. As MSDs result from chronic exposure to various risk factors, the accumulation of these exposures year after year in their work environment would explain a higher incidence among our participants [31]. In addition, a majority of our participants were overweight (47.5%) and 25.8% were obese. This would also tend to explain the high prevalence of MSDs among our study participants. Overweight, in addition to being a cardiovascular risk factor, has been shown to increase the risk of back pain due to the increased physical load exerted on articular discs and the musculoskeletal system of the spine in particular [32, 33].

MSDs were also frequently reported in the neck and knee regions with their prevalences being 42.4% and 29.1% respectively. These results are similar to those of Szeto et al. who found prevalences of 55.6% and 35% among bus drivers in Japan [34]). Magnusson et al. in their study of taxi drivers in Switzerland found the prevalence MSDs of the neck to be 40% [20]. Raanas et al. found a prevalence of 57.8% in the neck region among taxi drivers in Norway [19]. Chen et al. found the prevalence of MSDs of the knee to be 19% in Taiwan [35]. All these results are similar to ours, and from this uniformity one could deduce that the different regions of the body would be subjected to similar exposures irrespective of the professional driver’s horizon.

In our study, 62% of participants had a low physical activity level. Similarly, Marcelo et al. in their study in Brazil reported that 69.84% of taxi drivers had a physical activity level below the WHO recommendations which recommend a minimum of 150 min of moderate physical activity or 75 min of intense physical activity per week in order to derive substantial health benefits [36]. This low physical activity level among professional drivers could be explained by their heavy workload, making them unavailable for sporting activities. In our study, 56.3% of taxi drivers worked daily and 58.3% were employees. The status of employee does not confer much decision-making freedom regarding the worker’s time use; Marcelo et al. also found that a great number of taxi drivers in Brazil worked every day (43.6%) and when asked the reason for their low physical activity level, the majority of them mentioned the heavy workload [36].

The physical activity level was not significantly associated with MSDs. In contrast, Wang et al. reported that taxi drivers who had at least one hour of daily physical activity had less back pain than the others and this difference was significant (OR = 2 *p* < 0.001) [37]. Similarly, Raanaas et al. found that a low physical activity level was associated with a high frequency of MSDs among taxi drivers in Norway (OR = 1.99 CI = 1.24–3.19) [19]. This disparity in results can be explained by the difference in sample size of the different studies. Indeed, these two studies that found an association, were done on large sample sizes, one of which was 719 and the other 929. However, in our study where this association was not found, the sample size was 151. This could explain the difficulty in highlighting an association. Studies should be performed on larger sample sizes in order to empirically clarify the role of physical activity in the prevention of MSDs.

Job dissatisfaction was a factor independently associated with MSDs (OR = 2.1 CI = 1.1–3.9). Job dissatisfaction generates additional stress in the worker. Stress leads to an increase in muscle tone by acting on the nervous and endocrine systems. It also leads to the release of catecholamines which are vasoconstrictive and the release of inflammatory cytokines. All these mechanisms contribute to increase the biomechanical load of the musculoskeletal system while limiting the repair of micro-injuries due to a lack of blood supply and the presence of inflammation [28]. Zulkarnain et al. in a review of bus drivers showed that job dissatisfaction and perceived stress were factors frequently associated with MSDs [25]. In 2014, Abledu et al. found that those taxi drivers who perceived their work as stressful and reported being dissatisfied with their work had an increased prevalence of MSDs compared to others [OR = 2.3 (1.5–3.0) OR = 2.0 (1.3–3.6)] [29]. In Furthermore, work-related stress contributes to the high prevalence of cardiovascular events [38,39]. There is a need to think about how to address job dissatisfaction and other sources of stress and their solutions would be beneficial for better health among taxi drivers.

Although not statistically significant, the habit of frequent napping increased the risk of having MSDs (OR = 4.5). Napping in the taxi lead to poor postures. These poor postures lead to an excessive force exerted on the joints and the overload of adjacent muscles and tendons [38]. Raanaas et al. found that taxi drivers who reported having frequent naps in the taxi had a higher prevalence of MSDs (OR = 1.63 CI = 1.17–2.26) [19]. It would therefore be wise for taxi drivers to limit naps in the taxi for better musculoskeletal health. Similarly, job tenure greater than 12 years was associated with MSDs (OR = 2.5) and this association was close to significance (*p* = 0.059). The cumulative effect of years of exposure is thought to be the cause [31]. Similarly, Zulkarnain et al. found in a systematic review among the 14 types of professional drivers including taxi drivers that seniority in the profession was associated with a higher prevalence of MSDs [25]. Other associated factors such as prolonged working hours, the carrying of loads and perceived work-related stress have been found in other studies but not in ours. This would probably be explained by the size of our sample which did not allow us to unveil further statistically significant associations.

This study has certain limitations. The type of study carried out as well as our sample size did not allow us to properly assess the effect of professional, personal factors and further psychosocial factors in the incidence of musculoskeletal disorders.

## Conclusions

The overall prevalence of MSDs among taxi drivers in the city of Yaoundé was 86.8% over the past 12 months; the back region was the most affected with a prevalence of 72.8%; Job dissatisfaction was associated with high risk of MSDs. The majority of taxi drivers had a level of physical activity below the WHO recommendations; No significant association was found between physical activity level and MSDs. In order to better guide prevention strategies, analytical and longitudinal studies comprising a clinical assessment would be a better way to objectify the presence of MSDs, identify all its risk factors and their impact on the occurrence of MSDs.

## Data Availability

The datasets used and/or analysed during the current study are available from the corresponding author on reasonable request.

## Abbreviations

MSDs: Musculoskeletal disorders
GPAQ: Global physical activity questionnaire
WHO: Word health organization
